# Defined and Scalable Generation of Hepatocyte-like Cells from Human Pluripotent Stem Cells

**DOI:** 10.3791/55355

**Published:** 2017-03-02

**Authors:** Yu Wang, Sharmin Alhaque, Kate Cameron, Jose Meseguer-Ripolles, Baltasar Lucendo-Villarin, Hassan Rashidi, David C. Hay

**Affiliations:** ^1^MRC Centre for Regenerative Medicine, University of Edinburgh

**Keywords:** Developmental Biology, Issue 121, hepatocyte-like cells, recombinant laminins, human pluripotent stem cells, GMP-ready system, scale-up, drug screening, cell therapy

## Abstract

Human pluripotent stem cells (hPSCs) possess great value for biomedical research. hPSCs can be scaled and differentiated to all cell types found in the human body. The differentiation of hPSCs to human hepatocyte-like cells (HLCs) has been extensively studied, and efficient differentiation protocols have been established. The combination of extracellular matrix and biological stimuli, including growth factors, cytokines, and small molecules, have made it possible to generate HLCs that resemble primary human hepatocytes. However, the majority of procedures still employ undefined components, giving rise to batch-to-batch variation. This serves as a significant barrier to the application of the technology. To tackle this issue, we developed a defined system for hepatocyte differentiation using human recombinant laminins as extracellular matrices in combination with a serum-free differentiation process. Highly efficient hepatocyte specification was achieved, with demonstrated improvements in both HLC function and phenotype. Importantly, this system is easy to scale up using research and GMP-grade hPSC lines promising advances in cell-based modelling and therapies.

**Figure:**
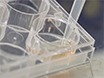


## Introduction

Primary human tissue and the derivative cell types are regularly used, both for cell-based screening and in the clinic. However, access to these cells is severely limited due to insufficient organ donation and loss of cell phenotypes post-isolation^1^. hPSCs represent a promising alternative to primary tissue and facilitate the generation of genetically defined and renewable human somatic cells. Hepatocyte-like cells (HLCs) derived from hPSCs have already shown promise in this field. HLCs resemble primary human hepatocytes in various aspects, including cell morphology, hepatocyte gene expression, metabolic function, and sensitivity to drugs and viruses^2^,^3^,^4^,^5^,^6^,^7^,^8^. In addition, the unlimited proliferation and self-renewal capacity of both research- and GMP-grade hPSCs facilitates their application^9^,^10^.

Over a decade of research has produced a number of efficient hepatocyte differentiation procedures^2^,^3^,^5^,^11^,^12^,^13^,^14^,^15^,^16^,^17^,^18^,^19^,^20^. However, most of these systems use undefined components and/or viral transduction to drive hepatocellular specification. To improve the reliability of the technology at scale, it is important to develop a robust hepatocyte differentiation system that is truly defined, xeno-free, and GMP-compatible.

Laminins (LNs) are important extracellular matrix proteins that can influence cell adhesion, proliferation, migration, and differentiation. Laminins are heterotrimeric glycoproteins composed of one α, one β, and one γ chain. Recently, recombinant human laminins have been produced and used in cell biology. LN-511 has been shown to support the maintenance of hPSCs^21^, while a mixture of LN-521 and E-cadherin allows clonal derivation and the expansion of human embryonic stem cells^22^. LN-111, on the other hand, supports the maintenance of hepatoblast-like cells derived from hPSCs^23^. However, before our report, laminins 521 and 111 had not been utilized to generate HLCs with mature characteristics from hPSCs^10^.

Here, we detail procedures for culturing hPSCs on LN-521 and differentiating them on either LN-521 or a blend of LN-521 and LN-111 (LN-521/LN-111). We optimized the differentiation protocol using single-cell seeding to generate a highly reproducible and homogenous monolayer of HLCs in many formats^14^. We believe that our defined differentiation system represents a simple and cost-effective method to manufacture active HLCs for application, representing a significant step forward in the field.

## Protocol

NOTE: Vendor information for all reagents used in this protocol has been listed in **Table 1**. All media/plates should be sterile and at least at room temperature when cells are to have direct contact with them.

### 1. Passaging Human Pluripotent Stem Cells (hPSCs) onto Laminin 521

NOTE: The cell passaging procedure described below is based on single cells and is ideal for the derivation of a homogenous population of hepatocyte-like cells from hPSCs. Colony plating is also applicable and has been described previously^24^.

1. Prepare laminin-coated plates as needed.
  1. Thaw the 100 µg/mL stock of recombinant laminin 521 (LN-521) at 4 °C.
  2. Dilute the thawed LN-521 in ice-cold 1x DPBS (with Ca^2+^/Mg^2+^) to make a 5 µg/mL solution.
  3. Add 1 mL of 5 µg/mL LN-521 solution to coat one well of a 6-well plate and rock to spread it evenly in the well.
  4. Incubate the plates in a 37 °C/5% CO_2_ cell culture incubator for 2 - 4 h for urgent use or in a 4 °C refrigerator overnight.
  5. Store the laminin-coated plates in a 4 °C refrigerator as required. Keep the plates on a flat surface and seal them to avoid evaporation and contamination. NOTE: Never let the coated wells dry out; top them up with extra 1x DPBS (with Ca^2+^/Mg^2+^) if required. Use the plates within 2 weeks.
2. Allow the required number of pre-coated plates to reach room temperature before use or incubate the plates at 37 °C for 0.5 - 1 h.
3. Carefully aspirate the coating LN-521 solution without damaging the coated surface. Note: It is critical not to damage the coated surface before seeding cells on it.
4. Immediately add 1 mL of pre-warmed-mTeSR1 medium supplemented with 10 µM Rho-associated kinase (ROCK) inhibitor Y27632 to one well of a 6-well plate. Leave the plate in the cell culture incubator to receive cells. NOTE: Never allow the laminin-coated wells to dry.
5. Aspirate the medium from well-maintained hPSCs at about 75% to 85% confluency. Wash the cells from one well of a 6-well plate once with 1 mL of room-temperature 1x DPBS (no Ca^2+^/Mg^2+^).
6. Add 0.5 mL of 1x Accutase to the cells and incubate at 37 °C for 6 - 8 min to dissociate the cells. NOTE: To check whether the digestion is long enough or not, gently tap the plate and check whether the cells can detach easily. If yes, then it is time to stop the enzymatic reaction; if not, extend the digestion for an extra 1 - 2 min.
7. Terminate the dissociation by adding 2 mL of fresh mTeSR1 medium supplemented with 10 µM Y27632 to the cells. Pipette up and down using a P1000 tip several times to make a single-cell suspension.
8. Count the viable cells using a hemocytometer. Use Trypan Blue to stain and exclude dead cells^14^
9. Calculate the total number of cells needed. For routine hPSC passaging, seed 4 x 10^5 ^to 5 x 10^5^ cells per well of a 6-well plate (*i.e.,* 4.21 x 10^4 ^to 5.26 x 10^4 ^per cm^2^). For passaging hPSCs for hepatocyte differentiation, seed 6.5 x 10^5 ^to 7.5 x 10^5^ (*i.e.,* 6.84 x 10^4^ to 7.89 x 10^4^ per cm^2^) cells per well of a 6-well plate. NOTE: The seeding density for each cell line might need minor optimization based on the empirical density given here for hepatic differentiation.
10. Transfer the needed cell suspension into a sterile 15-mL or 50-mL centrifuge tube and centrifuge at 115 x g for 3 min at room temperature.
11. Aspirate the supernatant slowly and then resuspend the cell pellet in fresh, warm mTeSR1 medium supplemented with 10 µM Rho-associated kinase (ROCK) inhibitor Y27632, using adequate medium to make the desired cell density. NOTE: The use of ROCK inhibitor is highly recommended in order to enhance cell attachment and survival rate.
12. Seed the cells to the prepared plates and rock them back and forth and side to side to evenly distribute the cells. NOTE: It is critical to ensure that cells are distributed evenly in the wells whether the plate is for routine cell culture or hepatocyte differentiation experimentation.
13. Place the plates in the cell incubator and maintain the cells at 37 °C/5% CO_2 _for 24 h to allow them to attach and recover.
14. Examine the cells the next day and withdraw ROCK inhibitor if cell-cell contact has been established. Maintain the cells in mTeSR1 medium for routine culture or switch to differentiation medium as needed. NOTE: If the cells were seeded with the mentioned density, the confluency should be ideal for routine maintenance or hepatocyte differentiation.

### 2. Differentiating hPSCs to Hepatocyte-like Cells on Recombinant Laminins

1. Prepare differentiation medium.
  1. Make human Activin A stock solution: dissolve human Activin A powder to make a 100 µg/mL stock solution in sterile 0.2% bovine serum albumin (BSA)/DPBS. Make small aliquots and store them at -20 °C. Use at 1:1,000.
  2. Make mouse Wnt 3a stock solution: dissolve mouse Wnt 3a powder to make a 10 µg/mL stock solution in sterile 0.2% BSA/DPBS. Make small aliquots and store them at -20 °C. Use at 1:200.
  3. Make human hepatocyte growth factor (HGF) stock solution: dissolve human HGF powder to make a 10 µg/mL stock solution in sterile 0.2% BSA/DPBS. Make small aliquots and store them at -20 °C. Use at 1:1,000.
  4. Make Oncostatin M (OSM) stock solution: dissolve Oncostatin M (OSM) powder to make a 20 µg/mL stock solution in sterile 0.2% BSA/DPBS. Make small aliquots and store them at -20 °C. Use at 1:1,000.
  5. Make 500 mL of endoderm-priming stock medium: 2% B27 supplement (50x, **minus vitamin A**) and 1% penicillin/streptomycin (final concentrations at 100 IU/mL and 100 µg/mL, respectively); top up to 500 mL using Roswell Park Memorial Institute 1640 (RPMI 1640) basal medium. NOTE: Store the stock at 4 °C and use within two weeks. Aliquot medium from the stock and add fresh Activin A and Wnt 3a (final concentrations at 100 ng/mL and 50 ng/mL, respectively) at each medium change.
  6. Make 500 mL of KSR/DMSO differentiation medium: 80% knockout DMEM (KO-DMEM), 20% knockout serum replacement (KSR), 0.5% GlutaMAX, 1% non-essential amino acids (NEAA), 0.1 mM beta-mercaptoethanol, 1% DMSO, and 1% penicillin/streptomycin (final concentrations at 100 IU/mL and 100 µg/mL, respectively). Filter under vacuum. Store at 4 °C and use within two weeks.
  7. Make 500 mL of HepatoZYME maturation medium: 1% GlutaMAX, 10 µM hydrocortisone 21-hemisuccinate sodium salt (HCC), and 1% penicillin/streptomycin (final concentrations at 100 IU/mL and 100 µg/mL, respectively); top up to 500 mL using HepatoZYME basal medium. NOTE: Store the stock at 4 °C and use within two weeks. Aliquot medium from the stock and add fresh HGF and OSM (final concentrations at 10 ng/mL and 20 ng/mL, respectively) for each medium change.
2. Seed hPSCs for hepatocyte differentiation on LN-521, as described in section 1. If LN-521/LN-111 is to be used as the substrate, coat the plates with LN-521 and LN-111 (1:3 ratio) at the final laminin concentration of 5 µg/mL; the rest treatment should be the same as pure LN-521-coated plates. NOTE: LN-521/LN-111 is not ideal for routine culture of hPSCs; it is only used for differentiation experiments.
3. Check the cell confluency 24 h after seeding. Initiate cellular differentiation once the cell confluency reaches about 40%. Remove the spent mTeSR1 medium and add fresh endoderm-priming medium supplemented with 100 ng/mL Activin A and 50 ng/mL Wnt 3a. Call this differentiation day 1. NOTE: It is highly recommended to initiate the differentiation the day after seeding the cells.
4. Change the endoderm-priming medium every 24 h for 3 days for human embryonic stem cells (hESCs). As for human induced pluripotent stem cells (hiPSCs), extend this stage for 2 more days to prime the cells to definitive endoderm-like cells, but use the endoderm-priming medium supplemented only with 100 ng/mL Activin A for these two days^12^. NOTE: To ensure successful endoderm specification, one can examine the expression of endodermal markers, such as FOXA2 and SOX17. According to immunofluorescence staining, over 80% of the derived cells are positive for both markers in our lab.
5. Switch to KSR/DMSO differentiation medium on day 4 (for hESCs)/day 6 (for hiPSCs). Change the medium daily for the first 3 days and then on the fifth day of this differentiation stage. NOTE: No feeding is needed on the fourth day of this differentiation stage. Use unsupplemented KO-DMEM to wash the cells once before medium change if there are many dead cells. To check whether the differentiation for this stage is successful or not, one can test the expression of hepatic progenitor cell markers, such as AFP, CK19, and HNF4A. Nearly 90% of the cells will be positive for these markers based on our experience.
6. After 5 days of the KSR/DMSO differentiation stage, switch to the HepatoZYME maturation stage. Wash the cells once with plain HepatoZYME basal medium after removing the KSR/DMSO medium. Add HepatoZYME maturation medium supplemented with 10 ng/mL HGF and 20 ng/mL OSM.
7. Change the medium every 48 h for 7 - 10 days, at which point the cells are ready for standard characterization or further use. NOTE: Hepatocyte marker expression examinations, metabolic function tests (such as cytochrome P450 activity), urea and albumin secretion tests, glycogen storage tests, and Indocyanine green (ICG) uptake tests are typical characterization methods. NOTE: The timeline of the differentiation protocol is shown in **Figure 5**. In our lab, we routinely check the derived HLCs' hepatocyte-specific marker expression levels, albumin secretion, and cytochrome P450 (CYP) 3A and 1A2 activity.

## Representative Results

### Hepatocellular Differentiation from hPSCs

One human embryonic stem cell line, H9, and one human induced pluripotent stem cell line, 33D6, were used for hepatocyte differentiation. The results in **Figures 1-3 **are from H9 cells, while those in **Figure 4** are from 33D6 cells. Single cells seeded onto laminins established cell-cell contact after 24 h. After the cells reached around 40% confluency, the differentiation process was initiated (**Figure 1A** and **Figure 4A**). On laminins (both LN-521 and LN-521/LN-111), these cells went through sequential morphological changes and gave rise to polarized HLCs (**Figure 1 **and **Figure 4A**).

### Hepatocyte-like Cell Characterization

Day 18 HLCs were collected and assessed for the expression of representative hepatocyte markers, *HNF4A* and *ALB *(**Figure 2A**). Immunostaining of day 18 HLCs showed that nearly 90% of the cells expressed HNF4α (**Figure 2B**). These cells polarized on laminins and exhibited a polygonal appearance, as marked by E-cadherin and F-actin expression (**Figure 2B**).

Cytochrome P450 (CYP) activity was also assessed. The CYP450s conduct an important metabolic function of hepatocytes. Day 18 HLCs derived on a gelatinous protein mixture, such as Matrigel, LN-521, or LN-521/LN-111, were tested for CYP3A activity. HLCs demonstrated significantly higher CYP3A activity on laminin substrates than on matrigel (**Figure 3**). Importantly, when compared to commercial human primary hepatocytes (HU1339) re-plated on these substrates, HLCs have nearly 10 times higher levels of CYP3A activity^10^.

The differentiation of hiPSCs was similar to that of hESCs. The cells exhibited sequential changes in appearance (**Figure 4A**). Derived HLCs expressed a key hepatocyte transcription factor, HNF4α (**Figure 4B**), and possessed CYP3A activity and secreted albumin (**Figure 4C **and** D**). Notably, HLCs derived from 33D6 displayed reduced CYP3A in comparison to H9 cells derived HLCs (**Figure 3**), but it was still comparable to human primary hepatocytes^10^. However, the albumin secretion of these HLCs was much lower than in primary hepatocytes^10^.


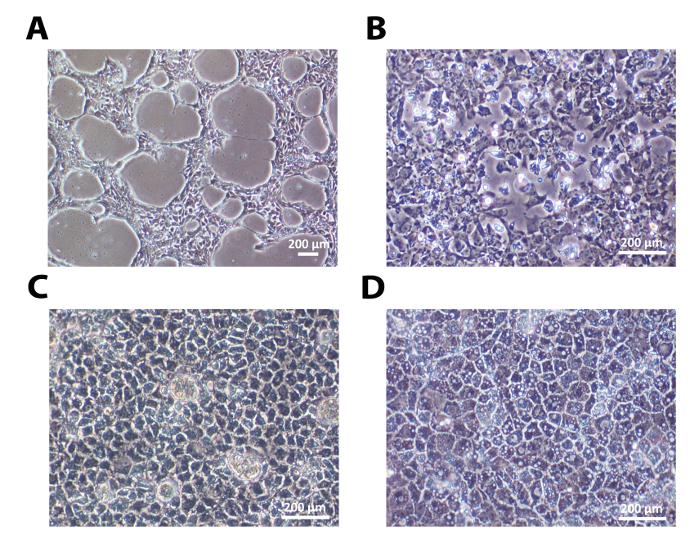
**Figure 1: The Sequential Morphological Changes during Hepatic Differentiation. (A)** Undifferentiated hESCs seeded as single cells reached around 40% confluency 24 h after seeding. **(B) **After priming, cells exhibited the typical endodermal morphology on day 4. **(C)** Upon reaching the hepatoblast-like stage, they showed a clear polygonal shape on day 9. **(D) **After the maturation stage, polarized HLCs were ready for further characterization shown here at day 18. Scale bar = 200 µm. Please click here to view a larger version of this figure.


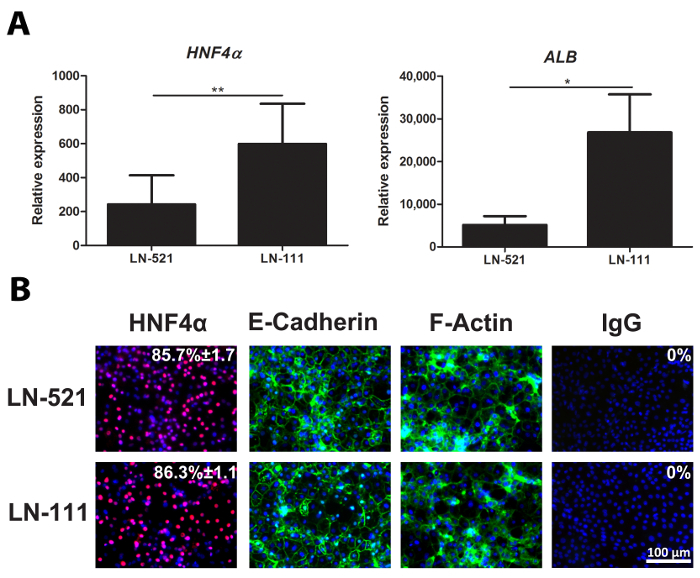
**Figure 2: Characterization of HLCs. (A) **Gene expression of hepatocyte-specific markers, *HNF4A *(left) and *ALB* (right). The expression level was analyzed using day 18 HLCs derived from hESCs on both LN-521 and LN-521/LN-111, and it was normalized to the housekeeping gene *GAPDH* and expressed relative to hESCs. The results represent three biological replicates, and the error bars represent standard deviation (SD). * p < 0.05, ** p < 0.01; unpaired t-test. **(B) **Protein expression of a key hepatic marker, HNF4α, and polarization markers, E-cadherin and F-actin. Day 18 HLCs on LN-521 and LN-521/LN-111 were stained for the above markers and counterstained with Hoechst 33342. A negative control was performed with the corresponding immunoglobulin G (IgG). The percentage of HNF4α-positive cells and the SD is shown. This was calculated from four random fields of view. Images were taken at 20X magnification. Scale bar = 100 µm. Please click here to view a larger version of this figure.


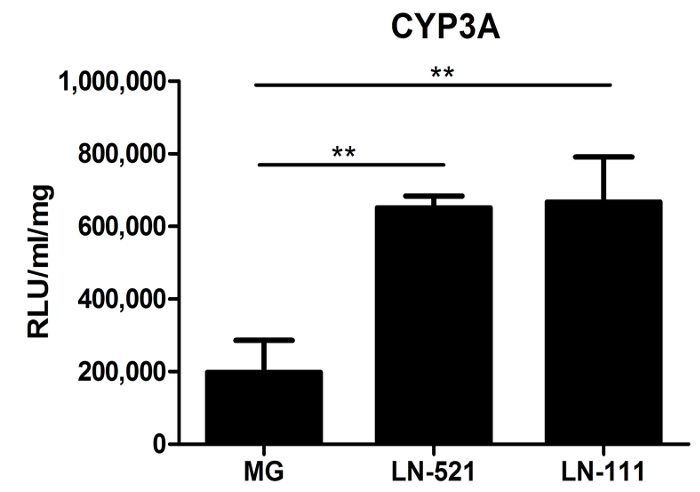
**Figure 3: Metabolic Function Characterization of HLCs. **Cytochrome P450 activity of CYP3A of cells cultured on Matrigel (MG), LN-521, or LN-521/LN-111 was tested. The data represent three biological replicates, and the error bars represent SD. ** p < 0.01; one-way ANOVA with Tukey's post-hoc test. Please click here to view a larger version of this figure.


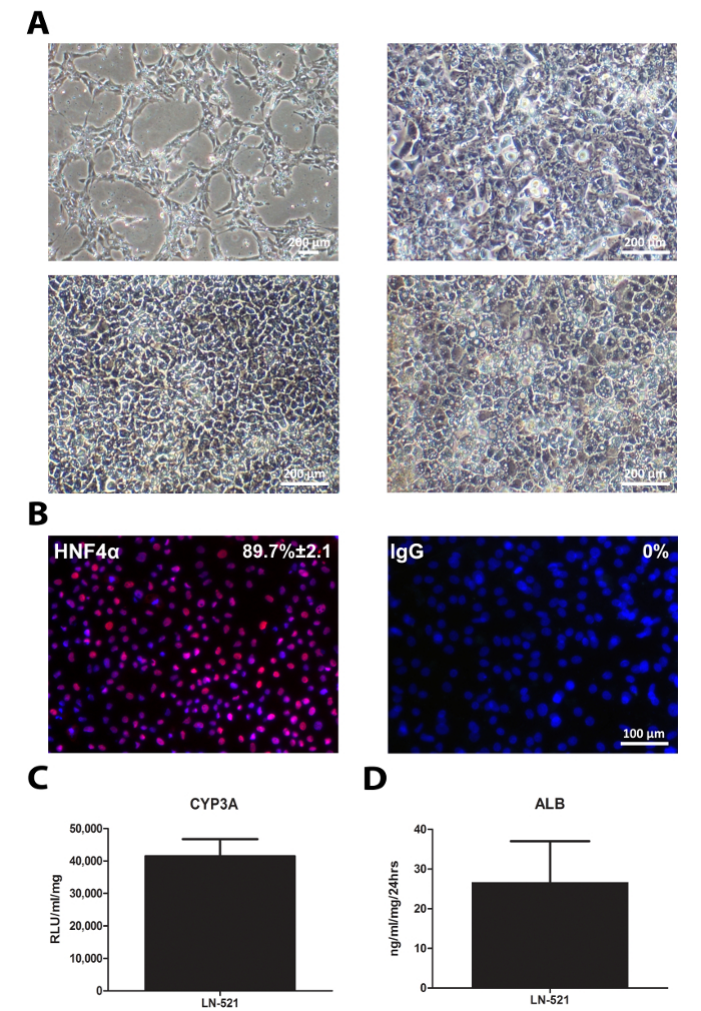
**Figure 4: Standard Characterization of HLCs. **hiPSCs cultured on LN-521 were differentiated into HLCs. Standard characterization tests were performed on day 17 HLCs. **(A)** The sequential morphology of the cells during hepatic differentiation; the time points shown represent cells on days 1, 4, 9, and 17. **(B) **Immunostaining of HNF4α expression. The percentage of positive cells and the SD is shown based on four random fields of view. Images were taken at 20X magnification. Scale bar = 100 µm. **(C)** CYP3A activity on day 17 HLCs. The data represent six biological replicates, and the error bar represents the SD. **(D) **Albumin secretion of derived HLCs over 24 h in culture. The data represent four biological replicates, and the error bar represents SD. Please click here to view a larger version of this figure.


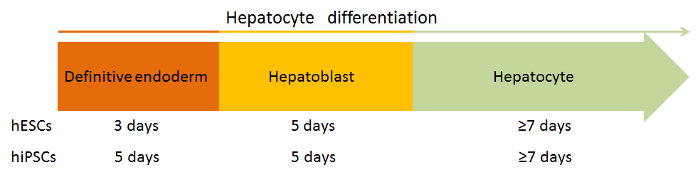
**Figure 5: Schematic Timeline of the Differentiation Protocol. **Please click here to view a larger version of this figure.  

## Discussion

To advance human pluripotent stem cell research and translational medicine, xeno-free systems that comply with current good manufacturing practice guidelines are required. Key to any differentiation process is the extracellular matrix (ECM). The ECM not only supports cell attachment, but also provides access to key signaling factors, influencing cell determination and phenotype^25^,^26^.

Laminins are multifunctional extracellular matrix proteins *in vivo*. In the liver, laminin secretion is crucial for liver regeneration after a partial hepatectomy^27^ and is required for hepatic progenitor cell maintenance^28^. The importance of laminins in liver maintenance and regeneration was the basis for testing commercially available recombinant human laminins in our hepatocyte differentiation system.

Superior hepatocyte differentiation was achieved on LN-521 and LN-521/LN-111 substrates when compared to Matrigel. Derived HLCs were clearly polarized and organized in the dish, and their cellular function was significantly improved when compared to their gelatinous protein mixture counterparts. Underlying these improvements was the downregulation of contaminating colon-, fibroblast- and stem cell-associated genes on the laminins, as well as a decrease in cell proliferation and migration-associated gene expression^10^.

In conclusion, the protocol described here generates hepatocyte-like cells that are closer in nature to adult human hepatocytes. The process is reproducible, amenable to automation, and can be cost-effectively scaled for application. Importantly, batch-to-batch variation has been significantly decreased in comparison to techniques that use Matrigel, resulting in an improved differentiation system for researchers within this field.

## Disclosures

Dr. David C. Hay is a co-founder and director of Stemnovate Limited.

## References

[B0] Forbes SJ, Gupta S, Dhawan A (2015). Cell therapy for liver disease: From liver transplantation to cell factory. J Hepatol.

[B1] Hay DC (2008). Highly efficient differentiation of hESCs to functional hepatic endoderm requires ActivinA and Wnt3a signaling. Proc Natl Acad Sci U S A.

[B2] Hay DC (2007). Direct differentiation of human embryonic stem cells to hepatocyte-like cells exhibiting functional activities. Cloning Stem Cells.

[B3] Medine CN (2013). Developing high-fidelity hepatotoxicity models from pluripotent stem cells. Stem Cells Transl Med.

[B4] Szkolnicka D (2014). Accurate prediction of drug-induced liver injury using stem cell-derived populations. Stem Cells Transl Med.

[B5] Zhou X (2014). Modulating innate immunity improves hepatitis C virus infection and replication in stem cell-derived hepatocytes. Stem Cell Reports.

[B6] Rashidi H, Alhaque S, Szkolnicka D, Flint O, Hay DC (2016). Fluid shear stress modulation of hepatocyte-like cell function. Arch Toxicol.

[B7] Szkolnicka D (2016). Reducing Hepatocyte Injury and Necrosis in Response to Paracetamol Using Noncoding RNAs. Stem Cells Transl Med.

[B8] Wang Y, Hay DC (2016). Mass production of stem cell derived human hepatocytes for experimental medicine. Expert Rev Gastroenterol Hepatol.

[B9] Cameron K (2015). Recombinant Laminins Drive the Differentiation and Self-Organization of hESC-Derived Hepatocytes. Stem Cell Reports.

[B10] Cai J (2007). Directed differentiation of human embryonic stem cells into functional hepatic cells. Hepatology.

[B11] Sullivan GJ (2010). Generation of functional human hepatic endoderm from human induced pluripotent stem cells. Hepatology.

[B12] Duan Y (2007). Differentiation and enrichment of hepatocyte-like cells from human embryonic stem cells in vitro and in vivo. Stem Cells.

[B13] Szkolnicka D, Farnworth SL, Lucendo-Villarin B, Hay DC (2014). Deriving functional hepatocytes from pluripotent stem cells. Curr Protoc Stem Cell Biol.

[B14] Rashid ST (2010). Modeling inherited metabolic disorders of the liver using human induced pluripotent stem cells. J Clin Invest.

[B15] Si-Tayeb K (2010). Highly efficient generation of human hepatocyte-like cells from induced pluripotent stem cells. Hepatology.

[B16] Touboul T (2010). Generation of functional hepatocytes from human embryonic stem cells under chemically defined conditions that recapitulate liver development. Hepatology.

[B17] Touboul T (2016). Stage-specific regulation of the WNT/beta-catenin pathway enhances differentiation of hESCs into hepatocytes. J Hepatol.

[B18] Mathapati S (2016). Small-Molecule-Directed Hepatocyte-Like Cell Differentiation of Human Pluripotent Stem Cells. Curr Protoc Stem Cell Biol.

[B19] Takayama K (2012). Efficient generation of functional hepatocytes from human embryonic stem cells and induced pluripotent stem cells by HNF4alpha transduction. Mol Ther.

[B20] Rodin S (2010). Long-term self-renewal of human pluripotent stem cells on human recombinant laminin-511. Nat Biotechnol.

[B21] Rodin S (2014). Clonal culturing of human embryonic stem cells on laminin-521/E-cadherin matrix in defined and xeno-free environment. Nat Commun.

[B22] Takayama K (2013). Long-term self-renewal of human ES/iPS-derived hepatoblast-like cells on human laminin 111-coated dishes. Stem Cell Reports.

[B23] Medine CN, Lucendo-Villarin B, Zhou W, West CC, Hay DC (2011). Robust generation of hepatocyte-like cells from human embryonic stem cell populations. J Vis Exp.

[B24] Sales VL (2006). Transforming growth factor-beta1 modulates extracellular matrix production, proliferation, and apoptosis of endothelial progenitor cells in tissue-engineering scaffolds. Circulation.

[B25] Taylor-Weiner H, Schwarzbauer JE, Engler AJ (2013). Defined extracellular matrix components are necessary for definitive endoderm induction. Stem Cells.

[B26] Martinez-Hernandez A, Delgado FM, Amenta PS (1991). The extracellular matrix in hepatic regeneration. Localization of collagen types I, III, IV, laminin, and fibronectin. Lab Invest.

[B27] Lorenzini S (2010). Characterisation of a stereotypical cellular and extracellular adult liver progenitor cell niche in rodents and diseased human liver. Gut.

